# Pleiotropy between neuroticism and physical and mental health: findings from 108 038 men and women in UK Biobank

**DOI:** 10.1038/tp.2016.56

**Published:** 2016-04-26

**Authors:** C R Gale, S P Hagenaars, G Davies, W D Hill, D C M Liewald, B Cullen, B W Penninx, International Consortium for Blood Pressure GWAS, CHARGE Consortium Aging and Longevity Group, D I Boomsma, J Pell, A M McIntosh, D J Smith, I J Deary, S E Harris

**Affiliations:** 1 Centre for Cognitive Ageing and Cognitive Epidemiology, University of Edinburgh, Edinburgh, UK; 2 Department of Psychology, University of Edinburgh, Edinburgh, UK; 3 MRC Lifecourse Epidemiology Unit, University of Southampton, Southampton General Hospital, Southampton, UK; 4 Division of Psychiatry, University of Edinburgh, Edinburgh, UK; 5 Institute of Health and Wellbeing, University of Glasgow, Glasgow, UK; 6 Department of Psychiatry, EMGO Institute for Health and Care Research and Neuroscience Campus Amsterdam, VU University Medical Center/GGZ inGeest, Amsterdam, The Netherlands; 7 Department of Biological Psychology, VU University Amsterdam, Amsterdam, The Netherlands; 8 Medical Genetics Section, University of Edinburgh Centre for Genomic and Experimental Medicine and MRC Institute of Genetics and Molecular Medicine, Western General Hospital, Edinburgh, UK

## Abstract

People with higher levels of neuroticism have an increased risk of several types of mental disorder. Higher neuroticism has also been associated, less consistently, with increased risk of various physical health outcomes. We hypothesised that these associations may, in part, be due to shared genetic influences. We tested for pleiotropy between neuroticism and 17 mental and physical diseases or health traits using linkage disequilibrium regression and polygenic profile scoring. Genetic correlations were derived between neuroticism scores in 108 038 people in the UK Biobank and health-related measures from 14 large genome-wide association studies (GWASs). Summary information for the 17 GWASs was used to create polygenic risk scores for the health-related measures in the UK Biobank participants. Associations between the health-related polygenic scores and neuroticism were examined using regression, adjusting for age, sex, genotyping batch, genotyping array, assessment centre and population stratification. Genetic correlations were identified between neuroticism and anorexia nervosa (*r*_g_=0.17), major depressive disorder (*r*_g_=0.66) and schizophrenia (*r*_g_=0.21). Polygenic risk for several health-related measures were associated with neuroticism, in a positive direction in the case of bipolar disorder, borderline personality, major depressive disorder, negative affect, neuroticism (Genetics of Personality Consortium), schizophrenia, coronary artery disease, and smoking (*β* between 0.009–0.043), and in a negative direction in the case of body mass index (*β*=−0.0095). A high level of pleiotropy exists between neuroticism and some measures of mental and physical health, particularly major depressive disorder and schizophrenia.

## Introduction

There is considerable evidence that the personality trait of neuroticism^1^—which describes stable individual differences in the tendency to experience negative emotions—has profound significance for public health.^2^ People who are higher in neuroticism have an increased risk of developing Axis I psychopathology, especially the common mental disorders such as mood, anxiety, somatoform and substance use disorders, and also schizophrenia, bipolar disorder and attention-deficit hyperactivity disorder (ADHD).^3, 4, 5, 6^ Higher neuroticism is associated with increased likelihood of diagnosis with Axis II personality disorders^7^ and with greater comorbidity between internalising disorders (such as major depression, generalised anxiety, panic disorders and phobias) and externalising disorders (such as alcohol and drug dependence, antisocial personality and conduct disorders).^8^ There is evidence that higher neuroticism is linked with risk of developing Alzheimer's disease.^9^ People who are higher in neuroticism tend to make greater use of mental health services, regardless of whether they have a mental disorder,^10^ perhaps because they are more likely to perceive a need for care.^11^ The estimated economic costs of neuroticism in terms of health-care use and absenteeism are massive.^12^ Much of these costs relate to reported chronic somatic conditions.^12^ Higher neuroticism has been linked with increased somatic complaints,^13, 14^ with perception of poorer health,^15, 16^ with future somatic multi-morbidity, as assessed by a count of reported chronic conditions,^17^ and with increased likelihood of reporting a range of physical health problems.^18^

Evidence that neuroticism is predictive of objectively assessed physical health is still relatively sparse and findings to date are often mixed. For example, whereas some prospective studies have found that higher neuroticism increases mortality from all causes^19^ or coronary heart disease,^20^ and is predictive of raised blood pressure^21^ or body mass index (BMI),^22^ others have found no such association.^23, 24, 25, 26^ In a pooled analysis of data from five cohorts, there was no consistent association between neuroticism and incidence of type 2 diabetes: higher neuroticism was linked with increased risk in one cohort, but not in others.^27^

Part of the explanation for associations between neuroticism and these various mental and physical health outcomes may be due to shared genetic influences. Twin and adoption studies suggest that genetic influences account for between a third and a half of individual differences in neuroticism.^1^ Many physical and mental illnesses and health-related measures also show moderate heritability.^28^ Twin studies have shown that there is considerable overlap between the genetic factors that influence variations in neuroticism and those that determine risk of depression and other internalising disorders.^29, 30^ It is now possible to test for such pleiotropy in associations between neuroticism and health outcomes using data from single nucleotide polymorphism (SNP) genotyping in unrelated individuals, making it possible to carry out much larger studies without the assumptions made by twin-study methods. A recent genome-wide association meta-analysis based on data from over 70 000 individuals found that neuroticism is influenced by many genetic variants of small effect, that is, a polygenic effect, that also influence the risk of major depressive disorder.^31^ Whether there is pleiotropy between neuroticism and other mental disorders or with physical health outcomes is unclear.

Testing for pleiotropy using SNP-based genetic data can be carried out in several ways. Linkage disequilibrium (LD) regression calculates genetic correlations between health measures using the summary results of genome-wide association studies (GWASs).^32^ It determines how much of the genetic influence on two traits/diseases is common to both. Polygenic risk scoring^33^ uses summary GWAS data for a given illness or health trait to test whether polygenic liability to that illness/trait is associated with phenotypes for that illness/trait (for example, neuroticism scores) or others measured in an independent sample. It allows the amount of variance in one trait/disease attributed to the polygenic score for a second trait/disease to be calculated. Polygenic risk of neuroticism was recently associated with major depressive disorder.^31^

In the present study we aimed to discover whether shared genetic aetiology explains part of the associations between neuroticism and various physical and mental health outcomes, all of which have been shown to be phenotypically correlated with neuroticism in at least one study. We used data on over 108 000 UK Biobank participants who completed a questionnaire on neuroticism and provided DNA for genome-wide genotyping. Using summary data from GWAS meta-analyses on 17 health-related measures, we tested for neuroticism-health pleiotropy using two complementary methods. First, we used LD score regression to derive genetic correlations between health-related measures and neuroticism. Second, we calculated the associations between polygenic risk scores for health-related measures and the neuroticism phenotype in UK Biobank participants.

## Materials and Methods

### Participants

The participants in this study took part in the baseline survey of UK Biobank.^34^ (http://www.ukbiobank.ac.uk). UK Biobank was set up as a resource for identifying determinants of disease in middle-aged and older people. Between 2006 and 2010, 502 655 community-dwelling people aged between 37 and 73 years and living in the United Kingdom were recruited to the study. They underwent assessments of cognitive and physical functions, mood and personality. They provided blood, urine and saliva samples for future analysis, completed questionnaires about their social backgrounds and lifestyle and agreed to have their health followed longitudinally. UK Biobank received ethical approval from the Research Ethics Committee (REC reference 11/NW/0382).

For the present study, genome-wide genotyping data were available on 112 151 individuals (58 914 female) aged 40–73 years (mean age=56.9 years, s.d.=7.9) after the quality control process (see below).

### Procedures

#### Neuroticism

Participants completed the Neuroticism scale of the Eysenck Personality Questionnaire-Revised (EPQ-R) Short Form.^35^ This scale has been concurrently validated in older people against two of the most widely used measures of neuroticism, taken from the International Personality Item Pool (IPIP) and the NEO-Five Factor Inventory (NEO-FFI); it correlated −0.84 with the IPIP-Emotional Stability scale and 0.85 with the NEO-FFI Neuroticism scale.^36^ A previous study found a high genetic correlation (0.91) between the EPQ-R Short Form Neuroticism scale and psychological distress assessed in a non-psychiatric population using the 30-item General Health Questionnaire.^37^

#### Genotyping and quality control

In all, 152 729 UK Biobank blood samples were genotyped using either the UK BiLEVE^38^ array (*N*=49 979) or the UK Biobank axiom array (*N*=102 750). A full description of the genotyping process is available in the Supplementary Materials. Quality control was performed by Affymetrix (Santa Clara, CA, USA), the Wellcome Trust Centre for Human Genetics and by the present authors; this included removal of participants based on missingness, relatedness, gender mismatch, non-British ancestry and other criteria, and is described in the Supplementary Materials.

#### Genome-wide association analyses in the UK Biobank sample

Genome-wide association analyses were performed on the neuroticism measure in order to use the summary results for LD regression. Details of the GWAS procedures are provided in the Supplementary Materials. Results from the GWAS are published elsewhere.^39^

#### Curation of summary results from GWAS consortia on health-related variables

In order to conduct LD score regression and polygenic profile score analyses between the UK Biobank neuroticism data and the genetic predisposition to mental and physical health outcomes, we gathered summary data from published meta-analyses on 17 health-related measures: 9 relating to mental health (ADHD, Alzheimer's disease, anorexia nervosa, bipolar disorder, borderline personality, major depressive disorder, negative affect, neuroticism (from the Genetics of Personality Consortium (GPC)) and schizophrenia) and 8 relating to physical health (systolic and diastolic blood pressure, BMI, coronary artery disease, longevity, rheumatoid arthritis, smoking status and type 2 diabetes). Details of these health-related variables, the consortia's websites, key references and number of subjects included in each consortium's GWAS are given in Supplementary Table 1.

### Statistical analysis

#### Computing genetic associations between neuroticism and health-related variables

We use two methods to compute genetic associations between neuroticism and the health-related variables, LD score regression and polygenic profile/risk scoring. Each provides a different metric to infer the existence of pleiotropy between pairs of traits. LD score regression was used to derive genetic correlations to determine the degree to which the polygenic architecture of a trait overlaps with that of another. Genetic correlations were also derived between the different physical and mental health measures, and neuroticism based on the results of the GWAS from the GPC by de Moor *et al.*^31^ A genetic correlation between the two neuroticism GWAS (UK Biobank and GPC) was also calculated. The polygenic risk score method was used to test the extent to which the polygenic information from GWASs of health-related variables could predict neuroticism in the UK Biobank participants. Both LD score regression and polygenic risk scores are dependent on the traits analysed being highly polygenic in nature, that is, where a large number of variants of small effect contribute towards phenotypic variation.^32, 40^ LD score regression was performed between the 14 health-related traits from GWAS consortia, as some measures did not pass thresholds to be included in LD score analysis, while the polygenic profile score analyses were performed on the complete set of 17 health-related traits from GWAS consortia.

#### LD score regression

In order to quantify the extent of pleiotropy between neuroticism, measured in UK Biobank, and the collated health traits, we used LD score regression.^32, 41^ This is a class of techniques that exploits the correlational structure of the SNPs found across the genome (we provide more details of LD score regression in the Supplementary Materials). Here we use LD score regression to derive genetic correlations between neuroticism and health-related measures using 14 large GWAS consortia data sets that enable pleiotropy of their health-related traits to be quantified with the neuroticism trait in UK Biobank. We followed the data-processing pipeline devised by Bulik-Sullivan *et al.*^32, 41^ described in more detail in the Supplementary Materials. In order to ensure that the genetic correlation for the Alzheimer's disease phenotype was not driven by a single locus or biased the fit of the regression model, a 500-kb region centred on the *APOE* locus was removed and this phenotype was re-run. This additional model is referred to in Table 1 as ‘Alzheimer's disease (500 kb)'.

#### Polygenic profiling

The UK Biobank genotyping data required recoding from numeric (1, 2) allele coding to standard ACGT format before being used in polygenic profile scoring analyses. This was achieved using a bespoke programme developed by one of the present authors (DCML), details of which are provided in the Supplementary Materials.

Polygenic profiles were created for 17 health-related phenotypes (Table 2 and Supplementary Table 2) in all genotyped participants using PRSice.^42^ This software calculates the sum of alleles associated with the phenotype of interest across many genetic loci, weighted by their effect sizes estimated from a GWAS of that phenotype in an independent sample. Before creating the scores, SNPs with a minor allele frequency <0.01 were removed, and clumping was used to obtain SNPs in linkage equilibrium with an *r*^2^<0.25 within a 200-bp window. Multiple scores were then created for each phenotype containing SNPs selected according to the significance of their association with the phenotype. The GWAS summary data for the 12 health-related phenotypes were used to create five polygenic profiles for each in the UK Biobank participants, at thresholds of *P*<0.01, *P*<0.05, *P*<0.1, *P*<0.5 and all SNPs. The most predictive threshold will be presented in the main tables of this paper. The full results, including all five thresholds, can be found in Supplementary Table 2.

Associations between the polygenic profiles and neuroticism were examined in linear regression models, adjusting for age at measurement, sex, genotyping batch and array, assessment centre and the first 10 genetic principal components to adjust for population stratification. We corrected for multiple testing across all polygenic profile scores at all significance thresholds for associations with neuroticism using the false discovery rate method.^43^ As neuroticism on average is higher in females and likely declines with age,^2^ age- (under and over 60 years old) and gender-stratified models were examined.

## Results

Within UK Biobank, 108 038 individuals with genotype data completed the Neuroticism scale of the EPQ-R Short Form. Their mean (s.d.) score for neuroticism was 4.02 (3.17).

The genetic correlation between neuroticism measured in the UK Biobank and neuroticism measured in the GPC is 1.0 (s.e.=0.11).

**Figure 1:**
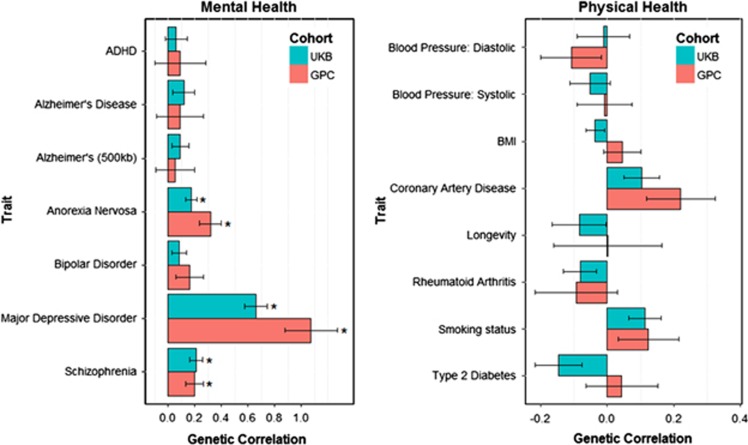
Barplot of genetic correlations (s.e.) calculated using linkage disequilibrium score regression between neuroticism in UK Biobank (UKB) and the Genetics of Personality Consortium (GPC), and mental and physical health measures from genome-wide association study consortia. *P<0.0033. ADHD, attention-deficit hyperactivity disorder; BMI, body mass index.

Table 1 shows the genetic correlations obtained using LD score regression between neuroticism in UK Biobank and neuroticism from the GPC, and the published GWAS results on the health-related traits. Neuroticism in UK Biobank showed significant positive genetic correlations with three traits, all related to mental health, namely major depressive disorder (*r*_g_=0.66), schizophrenia (*r*_g_=0.21) and anorexia nervosa (*r*_g_=0.17). The findings replicated when using Neuroticism from the GPC. There were no significant genetic correlations between neuroticism and either the other mental health-related traits (ADHD, Alzheimer's disease and bipolar disorder) or any of the physical health-related traits (systolic and diastolic blood pressure, BMI, coronary artery disease, type 2 diabetes, smoking status, rheumatoid arthritis or longevity). These results are shown graphically in Figure 1.

Table 2 shows the results of the polygenic risk scoring, using the most predictive threshold of the five that were created. Higher polygenic risk for six mental health-related traits, bipolar disorder, borderline personality, major depressive disorder, negative affect, neuroticism (calculated using summary data from the GPC) and schizophrenia, was significantly associated with higher levels of neuroticism in UK Biobank (standardised *β* between 0.017 and 0.043). There were no significant associations between neuroticism and polygenic risk for the other mental health-related traits examined, namely ADHD, Alzheimer's disease and anorexia nervosa. Polygenic risk scores for three physical health-related traits were significantly associated with neuroticism: higher polygenic risk for BMI was associated with lower levels of neuroticism (*β*=−0.0095, *P*=0.0015), higher polygenic risk for coronary artery disease was associated with higher levels of neuroticism (*β*=0.011, *P*=0.0003) and higher polygenic profile scores for smoking were associated with higher levels of neuroticism (*β*=0.17, *P*=2.48 × 10^−7^). No significant associations were found between polygenic risk for the other physical health-related traits (systolic and diastolic blood pressure, type 2 diabetes, rheumatoid arthritis or longevity) and neuroticism. To test whether the false discovery rate significant association between polygenic risk for coronary artery disease and neuroticism was confounded by individuals diagnosed with cardiovascular disease, 2717 individuals who had had a heart attack and 2468 individuals with angina were removed from the regression analysis. Our estimate of the association between polygenic risk of coronary artery disease and neuroticism was unchanged by this exclusion. The same applied for major depressive disorder (excluding 7494 individuals with a probable diagnosis of major depression or bipolar disorder^44^), where the estimate of the association showed little change. The complete polygenic risk score results, including all five thresholds, are shown in Supplementary Table 2.

Age- and gender-stratified analyses indicated there were no substantial differences by age or gender for 13 out of 17 traits. Four traits did show a potential age or gender effect. Polygenic risk for anorexia nervosa predicts neuroticism in males, but not in females. Polygenic risk for diastolic blood pressure predicts neuroticism only in females over 60 years of age. The strongest associations between polygenic risk for negative affect and smoking status, and neuroticism, are in females under 60 years of age. The full results for these stratified analyses can be found in Supplementary Table 3.

## Discussion

In this study of 108 038 men and women from UK Biobank who had been genotyped and assessed for neuroticism, we exploited the summary results of 17 large international GWAS consortia to examine whether there is pleiotropy between neuroticism and a range of physical and mental health outcomes using two methods, LD score regression and polygenic profile scoring. Summary results from two separate GWASs of neuroticism showed a genetic correlation of 1.0. Using summary data from both of these studies of neuroticism, the genetic correlations that were calculated with physical and mental health were very similar. As regards the six mental health outcomes that were investigated using both LD score regression and polygenic profile scoring, we found consistent evidence of pleiotropy between neuroticism and both major depression and schizophrenia with these methods, showing that, to a significant degree, the same genetic variants are responsible for the heritability of each pair of phenotypes and that genetic variants associated with major depression or schizophrenia in GWAS consortia are significantly predictive of variation in neuroticism in UK Biobank. There was some evidence for pleiotropy between neuroticism, and bipolar disorder, borderline personality, anorexia nervosa and negative affect on the basis of results from polygenic profile scoring or LD score regression, respectively, but the extent of these associations varied according to the method used. In all cases where there was a significant finding using one method but not the other, the direction of effect was the same using both methods. We found no evidence of pleiotropy between neuroticism and the other mental health outcomes examined—ADHD and Alzheimer's disease. Of the eight physical health outcomes studied, none showed evidence of pleiotropy with neuroticism on the basis of the genetic correlations obtained from LD score regression, but there was some indication of genetic overlap between neuroticism and coronary artery disease, smoking status and BMI. Higher polygenic risk for coronary artery disease and smoking status was significantly associated with higher levels of neuroticism, and polygenic risk for higher BMI was associated with lower levels of neuroticism. No associations were found between polygenic risk for the other physical health-related traits (systolic and diastolic blood pressure, rheumatoid arthritis, type 2 diabetes or longevity) and neuroticism.

Previous investigations into pleiotropy between neuroticism and mental health outcomes using polygenic risk profiling have found evidence of substantial shared genetic aetiology between neuroticism and major depression.^31, 45, 46^ Our observations in the present, much larger, sample confirm those findings and for, we believe, the first time quantify the extent to which the same genetic variants are responsible for the heritability in these two phenotypes, using an additional metric, LD score regression.^32^ The relatively high genetic correlation between neuroticism and major depression (*r*_g_=0.66), identified in our study, is similar to the genetic correlation identified in a previous twin study (*r*_g_=0.43).^30^ Our results also provide the first evidence, to our knowledge, that the phenotypic correlations found between neuroticism and schizophrenia^47, 48^ are due at least in part to genetic overlap. The extent of pleiotropy between anorexia nervosa or bipolar disorder and neuroticism has been unclear, though there is some, though limited, evidence to link both disorders phenotypically with neuroticism.^5, 49^ A previous study found no significant association between polygenic risk scores for neuroticism and bipolar disorder, but the sample size was small.^46^ In this much larger sample, higher polygenic risk scores for bipolar disorder were significantly predictive of higher neuroticism, but results of LD score regression showed little indication of genetic correlation between neuroticism and this disorder. We found a small but highly significant genetic correlation between neuroticism and anorexia (*r*_g_=0.17), but there was no association between polygenic risk scores for this condition and neuroticism. Although there is now considerable evidence that neuroticism is a risk factor for the development of Alzheimer's disease,^9^ there was no indication in our analyses that shared genes account for this link.

So far as we are aware, there have been no previous investigations of the extent of pleiotropy between neuroticism and physical health outcomes. This may be because, whereas there is considerable evidence for phenotypic associations between higher neuroticism and poorer self-rated health or greater somatic complaints,^13, 14, 15, 16, 17, 18^ fewer studies have examined neuroticism as a predictor of objectively measured physical health outcomes, and findings on such outcomes as coronary heart disease, blood pressure, BMI and all-cause mortality have been inconsistent.^19, 20, 21, 22, 23, 24, 25, 26^ Of the eight objectively measured physical health outcomes included in the current study, three showed evidence of a degree of genetic pleiotropy with neuroticism: coronary artery disease, smoking status and BMI. Neither demonstrated any measurable genetic correlation with neuroticism, but higher polygenic risk score for coronary artery disease was associated with higher neuroticism. This is consistent with the finding in pooled data from three cohorts that higher neuroticism was associated with increased mortality from coronary heart disease.^20^ Higher polygenic risk for BMI was associated with lower neuroticism. The direction of this association was unexpected. Findings on the phenotypic relationships between neuroticism and BMI have produced inconsistent results: one study showed higher neuroticism was associated with higher BMI,^22^ but another found no association.^25^

The chief strength of our study is the large sample size that permits powerful, robust tests of genetic association. Second, all the UK Biobank genetic data were processed at the same location and on the same platform. Finally, use of summary data from 17 large international GWAS consortia studies allowed us to perform a comprehensive examination of the degree of pleiotropy between neuroticism and a range of physical and mental health-related phenotypes, and to produce many of the first estimates of the genetic correlation between neuroticism and these phenotypes.

Our study also has some limitations. First, the GWAS studies we curated to carry out LD score regression and extract polygenic risk scores often involved meta-analyses of results from data sets with considerable heterogeneity in sample size, genome-wide imputation quality and measurement of phenotypes. With larger and more consistent independent data sets, it should be possible to use the polygenic risk scores to predict more variance in neuroticism. Second, we restricted the genotyped samples to individuals of white British ancestry in order to minimise any influence of population structure. Our results therefore need to be replicated in large samples with different genetic backgrounds as we did not have the power to model data from UK Biobank individuals of other ancestries. In the stratified analysis 13 out of 17 traits did not indicate a gender or age effect. These stratified results need to be interpreted with caution; they were mostly null, and no multiple testing correction was applied due to the high correlations between the different models. The four traits that did indicate potential gender or age effects need to be replicated in an independent sample, before any conclusions can be drawn from them.

We note that genetic correlations reflect the amount of the genetic influence on two traits that is common to both. This is independent of the heritability of either trait. Therefore, it is entirely possible for a trait to have a small proportion of variance accounted for by genetic variants, but to have a high genetic correlation with another trait. The amount of variance explained by each polygenic profile score is small, as would be expected by the fact that not all SNPs were genotyped, and those that were do not necessarily accurately tag the causal genetic variants. The polygenic risk score most predictive threshold varied between health traits, suggesting that the amount of shared genetic aetiology between neuroticism and each of the health traits differs. Although testing multiple thresholds may be deemed to increase the multiple testing problem, it should be noted that the SNPs included in each threshold are not independent. Also, at least for major depressive disorder and schizophrenia, pleiotropy was quantified using LD score regression, which involved only a single test per pair of phenotypes. The estimate of neuroticism variance explained by each polygenic profile should be considered as the minimum estimate of the variance explained. Owing to pruning SNPs in LD, the polygenic risk score method makes the assumption of a single causal variant being tagged in each LD block considered. If this assumption is not true for the phenotypes considered, the proportion of variance explained will be underestimated here.

In this large sample from UK Biobank, we aimed to discover whether shared genetic aetiology explained part of the associations between neuroticism and various physical and mental health outcomes. Our findings suggest that associations between neuroticism and several mental health outcomes including major depression, schizophrenia, bipolar disorder and anorexia nervosa are in part due to shared genetic influences. We found that polygenic risk scores for coronary artery disease, smoking and BMI were predictive of neuroticism scores. This large-scale mapping of the extent of pleiotropy between neuroticism and physical and mental health outcomes adds to our understanding of the cause of links between this important personality trait and later health.

## Figures and Tables

**Table 1:** Genetic correlations between neuroticism documented in UK Biobank and mental and physical health-related traits curated from GWAS consortia

*Trait Category*	*Traits from GWAS consortia*	*Neuroticism (UKB) (*n=*108 038)*			*Neuroticism (GPC) (*n=*63 661)*		
		r_*g*_	*s.e.*	P	r_*g*_	*s.e.*	P
Mental health	ADHD	0.060	0.082	0.4681	0.090	0.191	0.6371
	Alzheimer's disease	0.118	0.080	0.1378	0.089	0.177	0.6147
	Alzheimer's disease (500 kb)	0.091	0.063	0.1514	0.051	0.145	0.7262
	Anorexia nervosa	0.174	0.04	**2.36 × 10^−5^**	0.319	0.082	**9.56 × 10^−5^**
	Bipolar disorder	0.083	0.052	0.1096	0.162	0.101	0.1091
	Major depressive disorder	0.659	0.087	**2.75 × 10^−14^**	1.073	0.199	**7.15 × 10^−8^**
	Schizophrenia	0.212	0.050	**2.39 × 10^−5^**	0.198	0.068	**0.0033**
							
Physical health	Blood pressure: diastolic	−0.011	0.078	0.887	−0.108	0.091	0.2363
	Blood pressure: systolic	−0.051	0.060	0.3959	–0.008	0.083	0.925
	BMI	−0.036	0.028	0.1921	0.046	0.055	0.4077
	Coronary artery disease	0.104	0.053	0.0489	0.221	0.104	0.0331
	Longevity	−0.083	0.081	0.3011	0.002	0.163	0.9888
	Rheumatoid arthritis	−0.0816	0.05	0.103	−0.093	0.123	0.4463
	Smoking status	0.113	0.05	0.0205	0.124	0.090	0.1657
	Type 2 diabetes	−0.145	0.07	0.0389	0.044	0.107	0.6837

Abbreviations: ADHD, attention-deficit hyperactivity disorder; BMI, body mass index; GPC, Genetics of Personality Consortium; GWAS, genome-wide association study; UKB, UK Biobank.

Statistically significant *P*-values (after false discovery rate correction—*P*<0.0033) are shown in bold.

**Table 2:** Associations between polygenic risk scores for mental and physical health-related traits created from GWAS consortia summary data and neuroticism in UK Biobank participants, adjusted for age, sex, assessment centre, genotyping batch and array, and 10 principal components for population stratification

*Trait category*	*Traits from GWAS consortia*	*Neuroticism (*n=*108 038)*			
		*Threshold*	β	R^*2*^	P
Mental health	ADHD	0.05	0.0045	2.06 × 10^−5^	0.1292
	Alzheimer's disease	0.05	−0.0065	4.16 × 10^−5^	0.0312
	Anorexia nervosa	0.1	0.0054	2.87 × 10^−5^	0.0735
	Bipolar disorder	0.5	0.0171	0.0003	**1.71 × 10**^**−8**^
	Borderline personality	1	0.0150	0.0002	**5.43 × 10**^**−7**^
	Major depressive disorder	1	0.0357	0.0012	**1.23 × 10**^**−31**^
	Negative affect (anxiety)	1	0.00949	8.99 × 10^−5^	**1.55 × 10**^**−3**^
	Neuroticism (GPC)	0.5	0.0433	0.0020	**1.77 × 10**^**−47**^
	Schizophrenia	0.1	0.0359	0.0012	**7.88 × 10**^**−32**^
					
Physical health	Blood pressure: diastolic	0.1	−0.0047	2.20 × 10^−5^	0.1167
	Blood pressure: systolic	0.05	−0.0016	2.51 × 10^−6^	0.5969
	BMI	0.01	−0.0095	9.00 × 10^−5^	**0.0015**
	Coronary artery disease	0.1	0.0109	0.00012	**0.0003**
	Longevity	0.05	−0.0051	2.55 × 10^−5^	0.0917
	Rheumatoid arthritis	0.5	−0.0007	5.09 × 10^−5^	0.8266
	Smoking status	0.5	0.0167	0.0002	**2.48 × 10**^**−7**^
	Type 2 diabetes	0.01	−0.0031	9.67 × 10^−6^	0.2989

Abbreviations: ADHD, attention-deficit hyperactivity disorder; BMI, body mass index; GPC, Genetics of Personality Consortium; GWAS, genome-wide association study.

The associations between the polygenic risk scores and neuroticism with the largest effect size (threshold) are presented. Statistically significant *P*-values (after false discovery rate correction—*P*<0.0065) are shown in bold.
