# Supplementary material for: Pleiotropy between neuroticism and physical and mental health: findings from 108 038 men and women in UK Biobank
Source: Transl Psychiatry. 2016 Apr 26;6(4):e791–. doi: 10.1038/tp.2016.56 (PMC4872414; doi:10.1038/tp.2016.56)
Supplement: Supplementary Information [file tp201656x1.docx]

**Supplementary materials**

**Contents**

**Page 2:** Genotyping and quality control.

**Page 2**: Genome-wide association analyses (GWAS) in the UK Biobank sample.

**Page 2:** LD regression genetic correlation procedure.

**Page 3:** Polygenic profiling procedure.

**Page 4:** Sources of genetic results from genome-wide association studies (GWAS) consortia.

**Page 6:** Supplementary Table 1, on details of the sources of genetic results from genome-wide association studies (GWAS) consortia.

**Page 7:** Supplementary Table 2, showing complete polygenic risk score associations with neuroticism using all five thresholds.

**Page 10**: Supplementary Table 3a-d, showing age and gender stratified analyses for the polygenic risk score associations with neuroticism.

**Genotyping and Quality Control**

Genotyping was performed on 33 batches of ~4700 samples by Affymetrix, who also performed initial quality control of the genotyping data. Further details are available of the sample processing specific to the UK Biobank project (<http://biobank.ctsu.ox.ac.uk/crystal/refer.cgi?id=155583>) and the Axiom array (<http://media.affymetrix.com/support/downloads/manuals/axiom_2_assay_auto_workflow_user_guide.pdf>). Prior to the release of the UK Biobank genetic data a stringent QC protocol was applied, and performed at the Wellcome Trust Centre for Human Genetics (WTCHG); details of this process can be found at the following URL (<http://biobank.ctsu.ox.ac.uk/crystal/refer.cgi?id=155580>).

Prior to the analyses described in the main report, further quality control measures were applied by the present authors. Individuals were removed based on missingness, relatedness (KING estimated kinship co-efficient > 0·0442), gender mismatch, non-British ancestry (principal component analysis identified probable Caucasians within those individuals that were self-identified as British), and QC failure in the UK BiLEVE study. A sample of 112 151 individuals remained for further analyses. A minor allele frequency of maf < 1% filter was applied and only autosomal variants were used in this study (N = 705 516).

**Genome-wide association analyses (GWAS) in the UK Biobank sample**

An imputed dataset, including >70 million variants, was made available in which the UK Biobank interim release was imputed to a reference set which combined the UK10K haplotype and 1000 Genomes Phase 3 reference panels. Further details can be found at the following URL: <http://biobank.ctsu.ox.ac.uk/crystal/refer.cgi?id=157020>. A genome-wide association analysis of neuroticism was performed on the imputed dataset using SNPTest v2.5.1 (Marchini J, Howie B, Myers S, et al. A new multipoint method for genome-wide association studies via imputation of genotypes. Nat Genet 2007; 39: 906-913; SNPTEST v.2.5.1 can be found at the following URL: <https://mathgen.stats.ox.ac.uk/genetics_software/snptest/snptest.html#introduction>). An additive model was specified using the "frequentist 1" option. To account for genotype uncertainty we analysed the expected genotyped counts (dosages). Adjustments for age, sex, genotyping batch, genotyping array, assessment centre and 10 principal components were included. Prior to use in LD regression analyses the association results were filtered based on minor allele frequency (<0·1%) and imputation quality (<0·1).

**LD regression genetic correlation procedure**

Where available in the summary statistics provided, a MAF threshold of > 0·01 was applied. To control for imputation quality, only those SNPs found in the HapMap3 with 1000 Genomes EUR with a MAF > 0·05 were included (integrated_phase1_v3.20101123). Next, indels and structural variants were removed along with strand-ambiguous SNPs. Finally, genome-wide significant SNPs were removed, as were SNPs with very large effect sizes (χ^2^ > 80), as the presence of outliers can increase the standard error in a regression model. LD scores and weights for use with the GWAS of European ancestry were downloaded from the Broad institute (http://www.broadinstitute.org/~bulik/eur_ldscores/). Data sets demonstrating a heritability Z-score (h2z ) > 4 and a mean χ2 statistic of > 1.02 were included. All traits, except negative affect and borderline personality, exceeded these thresholds. An unconstrained intercept was used in the regression model as it was not possible to quantify the degree of sample overlap between the genome-wide association analyses used here.

**Polygenic profiling procedure**

The genetic data files (.map and .ped files) supplied from Biota (the UK Biobank online repository) were unsuitable for use in the polygenic profile analyses as the .ped allele coding used a 1, 2 numeric allele encode rather than the standard ACGT encode format. In order to enable the analysis, the .ped files were recoded to the standard encode format. To achieve this, a bespoke programme was developed to create new files using a lookup-substitution method. A fast-in-memory lookup string hash table was created to hold the SNP-ID, along with the allele identifiers for the SNP. A simple loop then performed serialised lookups based on string position, to create an associated string with the correct ACGT encode. This was then appended to the six mandatory data fields extracted from initial string. In order to maximise performance and enable timely completion of the lookup-substitution, these loops were run in parallel threads in a standard multiprocessor environment.

**Sources of genetic results from genome-wide association consortia**

**CARDIoGRAM**

Coronary artery disease data have been contributed by CARDIoGRAMplusC4D investigators.

**CHARGE-Aging and Longevity**

Longevity data have been provided by the CHARGE-Aging and Longevity consortium. Longevity was defined as reaching age 90 years or older. Genotyped participants who died between the ages of 55 and 80 years were used as the control group. There were 6036 participants who achieved longevity and 3757 participants in the control group across participating studies in the discovery meta-analysis.

Broer L, Buchman AS, Deelen J, Evans DS, Faul JD, Lunetta KL, Sebastiani P, Smith JA, Smith AV, Tanaka T, Yu L, Arnold AM, Aspelund T, Benjamin EJ, De Jager PL, Eirkisdottir G, Evans DA, Garcia ME, Hofman A, Kaplan RC, Kardia SL, Kiel DP, Oostra BA, Orwoll ES, Parimi N, Psaty BM, Rivadeneira F, Rotter JI, Seshadri S, Singleton A, Tiemeier H, Uitterlinden AG, Zhao W, Bandinelli S, Bennett DA, Ferrucci L, Gudnason V, Harris TB, Karasik D, Launer LJ, Perls TT, Slagboom PE, Tranah GJ, Weir DR, Newman AB, van Duijn CM and Murabito JM. **GWAS of Longevity in CHARGE Consortium Confirms APOE and FOXO3 Candidacy**. *J Gerontol A Biol Sci Med Sci*. 2015;70:110-8.

*Acknowledgments*

The CHARGE Aging and Longevity working group analysis of the longevity phenotype was funded through the individual contributing studies. The working group thanks all study participants and study staff**.**

**DIAGRAM**

Type 2 diabetes data were obtained from the DIAGRAM consortium.

**International Consortium of Blood Pressure (ICBP)**

Blood pressure data were provided by ICBP.

**GIANT**

BMI data was obtained from the GIANT consortium.

**International Genomics of Alzheimer’s Project (IGAP)**

Alzheimer’s disease data were obtained from (IGAP)

*Material and methods*

International Genomics of Alzheimer's Project (IGAP) is a large two-stage study based upon genome-wide association studies (GWAS) on individuals of European ancestry. In stage 1, IGAP used genotyped and imputed data on 7 055 881 single nucleotide polymorphisms (SNPs) to meta-analyse four previously-published GWAS datasets consisting of 17 008 Alzheimer's disease cases and 37 154 controls (The European Alzheimer's disease Initiative – EADI the Alzheimer Disease Genetics Consortium – ADGC The Cohorts for Heart and Aging Research in Genomic Epidemiology consortium – CHARGE The Genetic and Environmental Risk in AD consortium – GERAD). In stage 2, 11 632 SNPs were genotyped and tested for association in an independent set of 8572 Alzheimer's disease cases and 11 312 controls. Finally, a meta-analysis was performed combining results from stages 1 & 2.

*Acknowledgments*

We thank the International Genomics of Alzheimer's Project (IGAP) for providing summary results data for these analyses. The investigators within IGAP contributed to the design and implementation of IGAP and/or provided data but did not participate in analysis or writing of this report. IGAP was made possible by the generous participation of the control subjects, the patients, and their families. The i–Select chips was funded by the French National Foundation on Alzheimer's disease and related disorders. EADI was supported by the LABEX (laboratory of excellence program investment for the future) DISTALZ grant, Inserm, Institut Pasteur de Lille, Université de Lille 2 and the Lille University Hospital. GERAD was supported by the Medical Research Council (Grant n° 503480), Alzheimer's Research UK (Grant n° 503176), the Wellcome Trust (Grant n° 082604/2/07/Z) and German Federal Ministry of Education and Research (BMBF): Competence Network Dementia (CND) grant n° 01GI0102, 01GI0711, 01GI0420. CHARGE was partly supported by the NIH/NIA grant R01 AG033193 and the NIA AG081220 and AGES contract N01–AG–12100, the NHLBI grant R01 HL105756, the Icelandic Heart Association, and the Erasmus Medical Center and Erasmus University. ADGC was supported by the NIH/NIA grants: U01 AG032984, U24 AG021886, U01 AG016976, and the Alzheimer's Association grant ADGC–10–196728.

**Psychiatric Genetics Consortium**

Schizophrenia, bipolar disorder, major depressive disorder, and ADHD data were obtained from the Psychiatric Genetics Consortium.

**Genetic Consortium for Anorexia nervosa**

Anorexia nervosa data were obtained from the Genetic Consortium for Anorexia nervosa.

**Genetics of Personality Consortium**

Neuroticism data were obtained from the Genetics for Personality Consortium.

**Tobacco and Genetics Consortium**

Data on smoking status were obtained from the Tobacco and Genetics Consortium.

**Rheumatoid Arthritis**

Rheumatoid arthritis data were obtained from http://www.broadinstitute.org/ftp/pub/rheumatoid_arthritis/Stahl_etal_2010NG/

**Negative Affect**Maciej Trzaskowski and Robert Plomin kindly contributed negative affect data.

**NESDA & NTR**

Borderline personality data were obtained from NESDA and NTR.

*Acknowledgement*

Funding was obtained from the Netherlands Organization for Scientific Research (NWO) and MagW/ZonMW grants 904-61-090, 985-10-002, 912-10-020, 904-61-193,480-04-004, 463-06-001, 451-04-034, 400-05-717, Addiction-31160008, Middelgroot-911-09-032, Spinozapremie 56-464-14192, Center for Medical Systems Biology (CSMB, NWO Genomics), NBIC/BioAssist/RK(2008.024), Biobanking and Biomolecular Resources Research Infrastructure (BBMRI –NL, 184.021.007). VU University’s Institute for Health and Care Research (EMGO+) and Neuroscience Campus Amsterdam (NCA), University Medical Center Groningen, Leiden University Medical Center; the European Science Foundation (ESF, EU/QLRT-2001-01254), the European Community's Seventh Framework Program (FP7/2007-2013), ENGAGE (HEALTH-F4-2007-201413); the European Science Council (ERC Advanced, 230374), Rutgers University Cell and DNA Repository (NIMH U24 MH068457-06), the Avera Institute, Sioux Falls, South Dakota (USA) and the National Institutes of Health (NIH, R01D0042157-01A, MH081802, Grand Opportunity grants 1RC2 MH089951 and 1RC2 MH089995). Part of the genotyping and analyses were funded by the Genetic Association Information Network (GAIN) of the Foundation for the National Institutes of Health. Computing was supported by BiG Grid, the Dutch e-Science Grid, which is financially supported by NWO.

**Supplementary Table 1**

Sources of genetic results from genome-wide association consortia

| **Phenotype** | **Consortium** | **URL** | **Reference** | **No. of individuals in GWAS** |
| --- | --- | --- | --- | --- |
| Coronary Artery Disease | CARDIoGRAM | http://www.cardiogramplusc4d.org/downloads/ | Schunkert et al. Nat Genet 2011; 43: 333-338. PMID: 21378990 | 22 233 cases  64 762 controls |
| Type 2 diabetes | DIAGRAM | http://diagram-consortium.org/downloads.html | Morris et al. Nat Genet 2012; 44: 981-990. PMID: 22885922 | 12 171 cases  56 862 controls |
| ADHD | Psychiatric Genetics Consortium (PGC) | https://www.med.unc.edu/pgc/downloads | Cross-Disorder Group of the Psychiatric Genomics Consortium. Lancet 2013; 381: 1371-1379. PMID: 23453885 | 1947 trio cases 1947 trio pseudocontrols, 840 cases  688 controls |
| Alzheimer's disease | International Genomics of Alzheimer’s Project (IGAP) | http://www.pasteur-lille.fr/en/recherche/u744/igap/igap_download.php | Lambert et al. Nat Genet 2013; 45: 1452-1458. PMID: 24162737 | 17 008 cases  37 154 controls |
| Anorexia nervosa | Genetic Consortium for Anorexia Nervosa (GCAN) | http://www.med.unc.edu/pgc/downloads | Boraska, Vesna, et al. Molecular psychiatry 2014; 19(10): 1085-1094. PIMD: 24514567 | 2907 cases 14860 controls |
| Bipolar disorder | Psychiatric Genetics Consortium (PGC) | https://www.med.unc.edu/pgc/downloads | Psychiatric GWAS Consortium Bipolar Disorder Working Group. Nat Genet 2011; 43: 977-983. PMID: 21926972 | 7481 cases  9250 controls |
| Major depressive disorder | Psychiatric Genetics Consortium (PGC) | https://www.med.unc.edu/pgc/downloads | Major Depressive Disorder Working Group of the Psychiatric GWAS Consortium. Mol Psychiatr 2013; 18: 497-511. PMID: 22472876 | 9240 cases  9519 controls |
| Schizophrenia | Psychiatric Genetics Consortium (PGC) | https://www.med.unc.edu/pgc/downloads | Schizophrenia Working Group of the Psychiatric Genomics Consortium. Nature 2014; 511: 421-427. PMID: 25056061 | 36 989 cases  113 075 controls |
| BMI | GIANT | http://www.broadinstitute.org/collaboration/ giant/index.php/GIANT_  consortium_data_files | Locke et al. Nature 2015; 518: 197-206. PMID: 25673413 | 339 224 |
| Diastolic blood pressure | International Consortium of Blood Pressure (ICBP) |  | Ehret et al. (2011) Nature 478, 103-109. PMID: 21909115 | 69 395 |
| Longevity | CHARGE-Aging and Longevity |  | Broer et al. J Gerontol A Biol Sci Med Sci 2015; 70: 110-118. PMID: 25199915 | 6036 cases  3757 controls |
| Systolic blood pressure | International Consortium of Blood Pressure (ICBP) |  | Ehret et al. Nature 2011; 478: 103-109. PMID: 21909115 | 69 395 |
| Neuroticism | Genetics of Personality Consortium | http://www.tweelingenregister.org/GPC/ | De Moor et al. JAMA Psychiatry, 72(7): 642-650. PMID: 25993607 | 63 661 |
| Negative Affect |  |  | Trzaskowski et al. PLOS One 2013; 8(4) | 2810 cases |
| Rheumatoid Arthritis | - | http://www.broadinstitute.org/ftp/pub/rheumatoid_arthritis/Stahl_etal_2010NG/ | Stahl et al. Nat Genet 2010; 42:508-14. PMID: 20453842 | 5539 cases  20 169 controls |
| Smoking Status | Tobacco and Genetics Consortium | https://www.med.unc.edu/pgc/files/resultfiles/tag.evrsmk.tbl.gz | Tobacco and Genetics Consortium. Nat Genet 2010; 42(5): 441-447. PMID: 20418890 | 74 053 |
| Borderline Personality | NESDA and NTR |  | Lubke et al. Mol Psych 2015; 19:923-929 PMID: 23979607 | 7125 |

**Supplementary Table 2**
Associations between polygenic profiles of health related traits, and neuroticism controlling for age, sex, assessment centre, genotyping batch and array, and ten principal components for population structure. Statistically significant values (after False Discovery Rate correction, P < 0.0065) are shown in bold.

|  |  | **Neuroticism** | | | | |
| --- | --- | --- | --- | --- | --- | --- |
|  |  | Threshold | β | SE | R2 | p |
| **Mental health** | ADHD | 0.01 | -0.00287 | 0.002994 | 8.26×10^-6^ | 0.337012 |
|  |  | 0.05 | 0.004543 | 0.002994 | 2.06×10^-5^ | 0.129231 |
|  |  | 0.1 | 0.002974 | 0.002994 | 8.84×10^-6^ | 0.32062 |
|  |  | 0.5 | 0.003187 | 0.002996 | 1.01×10^-5^ | 0.287361 |
|  |  | 1 | 0.00351 | 0.002996 | 1.23×10^-5^ | 0.241329 |
|  | Alzheimer's Disease | 0.01 | -0.00139 | 0.002997 | 1.92×10^-6^ | 0.643781 |
|  |  | 0.05 | -0.00647 | 0.003002 | 4.16×10^-5^ | 0.031179 |
|  |  | 0.1 | -0.00523 | 0.003004 | 2.72×10^-5^ | 0.081515 |
|  |  | 0.5 | -0.00022 | 0.003008 | 4.70×10^-8^ | 0.942228 |
|  |  | 1 | 0.000818 | 0.003008 | 6.63×10^-7^ | 0.785614 |
|  | Anorexia Nervosa | 0.01 | 0.004731 | 0.002995 | 2.24×10^-5^ | 0.114197 |
|  |  | 0.05 | 0.003593 | 0.002995 | 1.29×10^-5^ | 0.230219 |
|  |  | 0.1 | 0.005362 | 0.002996 | 2.87×10^-5^ | 0.073496 |
|  |  | 0.5 | 0.000139 | 0.003001 | 1.93×10^-8^ | 0.963021 |
|  |  | 1 | -0.00112 | 0.00301 | 1.23×10^-6^ | 0.710872 |
|  | Bipolar Disorder | 0.01 | 0.012695 | 0.003 | 0.00016 | **2.33×10^-5^** |
|  |  | 0.05 | 0.015862 | 0.00301 | 0.000249 | **1.37×10^-7^** |
|  |  | 0.1 | 0.017013 | 0.003016 | 0.000285 | **1.70×10^-8^** |
|  |  | 0.5 | 0.017078 | 0.003028 | 0.000285 | **1.71×10^-8^** |
|  |  | 1 | 0.016886 | 0.003029 | 0.000278 | **2.50×10^-8^** |
|  | Borderline Personality | 0.01 | 0.012467 | 0.002995 | 0.000155 | **3.15×10^-5^** |
|  |  | 0.05 | 0.011175 | 0.002996 | 0.000125 | **0.000192** |
|  |  | 0.1 | 0.01116 | 0.002997 | 0.000124 | **0.000196** |
|  |  | 0.5 | 0.014967 | 0.002999 | 0.000223 | **6.02×10^-7^** |
|  |  | 1 | 0.015028 | 0.002999 | 0.000225 | **5.43×10^-7^** |
|  | Major Depressive Disorder | 0.01 | 0.017178 | 0.003003 | 0.000293 | **1.07×10^-8^** |
|  |  | 0.05 | 0.024092 | 0.003021 | 0.000569 | **1.54×10^-15^** |
|  |  | 0.1 | 0.028489 | 0.00303 | 0.000791 | **5.51×10^-21^** |
|  |  | 0.5 | 0.034745 | 0.003051 | 0.001161 | **4.92×10^-30^** |
|  |  | 1 | 0.035716 | 0.003051 | 0.001226 | **1.23×10^-31^** |
|  | Negative affect (anxiety) | 0.01 | 0.004815 | 0.002994 | 2.32×10^-5^ | 0.107844 |
|  |  | 0.05 | 0.004631 | 0.002994 | 2.14×10^-5^ | 0.121922 |
|  |  | 0.1 | 0.004652 | 0.002995 | 2.16×10^-5^ | 0.120399 |
|  |  | 0.5 | 0.008842 | 0.002997 | 7.80×10^-5^ | **0.003171** |
|  |  | 1 | 0.009487 | 0.002997 | 8.98×10^-5^ | **0.001546** |
|  | Neuroticism (GPC) | 0.01 | 0.026405 | 0.003 | 0.0007 | **1.38**×10^-^**^18^** |
|  |  | 0.05 | 0.033051 | 0.002995 | 0.001097 | **2.66**×10^-^**^28^** |
|  |  | 0.1 | 0.037028 | 0.002995 | 0.001376 | **4.30**×10^-^**^35^** |
|  |  | 0.5 | 0.043349 | 0.002994 | 0.001885 | **1.77**×10^-^**^47^** |
|  |  | 1 | 0.043267 | 0.002994 | 0.001877 | **2.71**×10^-^**^47^** |
|  | Schizophrenia | 0.01 | 0.029828 | 0.00302 | 0.000873 | **5.32×10^-23^** |
|  |  | 0.05 | 0.034372 | 0.003046 | 0.001139 | **1.66×10^-29^** |
|  |  | 0.1 | 0.035914 | 0.003058 | 0.001234 | **7.88×10^-32^** |
|  |  | 0.5 | 0.033529 | 0.003084 | 0.001058 | **1.63×10^-27^** |
|  |  | 1 | 0.033817 | 0.003086 | 0.001075 | **6.23×10^-28^** |
| **Physical health** | Blood Pressure: Diastolic | 0.01 | 0.003944 | 0.002995 | 1.55×10^-5^ | 0.187925 |
|  |  | 0.05 | -0.0033 | 0.002998 | 1.08×10^-5^ | 0.271665 |
|  |  | 0.1 | -0.00471 | 0.003002 | 2.20×10^-5^ | 0.116712 |
|  |  | 0.5 | -0.00294 | 0.003004 | 8.60×10^-6^ | 0.327081 |
|  |  | 1 | -0.00258 | 0.003003 | 6.60×10^-6^ | 0.39054 |
|  | Blood Pressure: Systolic | 0.01 | -0.00157 | 0.002996 | 2.45×10^-6^ | 0.601327 |
|  |  | 0.05 | -0.00159 | 0.002999 | 2.51×10^-6^ | 0.596891 |
|  |  | 0.1 | -0.00046 | 0.003 | 2.12×10^-7^ | 0.877804 |
|  |  | 0.5 | 0.000151 | 0.003005 | 2.26×10^-8^ | 0.959928 |
|  |  | 1 | -0.00124 | 0.003005 | 1.52×10^-6^ | 0.68005 |
|  | BMI | 0.01 | -0.00949 | 0.002995 | 9.00×10^-5^ | **0.001529** |
|  |  | 0.05 | -0.00857 | 0.002996 | 7.33×10^-5^ | **0.004219** |
|  |  | 0.1 | -0.00816 | 0.002997 | 6.64×10^-5^ | 0.006459 |
|  |  | 0.5 | -0.0049 | 0.003 | 2.39×10^-5^ | 0.102193 |
|  |  | 1 | -0.00508 | 0.003 | 2.57×10^-5^ | 0.090145 |
|  | Coronary Artery Disease | 0.01 | 0.005668 | 0.002994 | 3.21×10^-5^ | 0.058318 |
|  |  | 0.05 | 0.009317 | 0.002995 | 8.67×10^-5^ | **0.001866** |
|  |  | 0.1 | 0.010949 | 0.002995 | 0.00012 | **0.000257** |
|  |  | 0.5 | 0.010614 | 0.002996 | 0.000112 | **0.000396** |
|  |  | 1 | 0.010072 | 0.002996 | 0.000101 | **0.000776** |
|  | Longevity | 0.01 | -0.00181 | 0.003014 | 3.24×10^-6^ | 0.547298 |
|  |  | 0.05 | -0.00511 | 0.003028 | 2.55×10^-5^ | 0.091694 |
|  |  | 0.1 | -0.00093 | 0.002996 | 8.72×10^-7^ | 0.755097 |
|  |  | 0.5 | -0.00261 | 0.003001 | 6.78×10^-6^ | 0.38437 |
|  |  | 1 | -0.00217 | 0.003001 | 4.68×10^-6^ | 0.470043 |
|  | Rheumatoid Arthritis | 0.01 | 0.000553 | 0.002998 | -5.10E-05 | 0.853539 |
|  |  | 0.05 | -0.00065 | 0.003001 | -5.09E-05 | 0.829678 |
|  |  | 0.1 | -0.00013 | 0.003005 | -5.13E-05 | 0.964768 |
|  |  | 0.5 | -0.00066 | 0.00302 | -5.09E-05 | 0.826638 |
|  |  | 1 | -0.00058 | 0.003022 | -5.10E-05 | 0.846755 |
|  | Smoking Status | 0.01 | 0.003084 | 0.00304 | -4.21E-05 | 0.310425 |
|  |  | 0.05 | 0.014977 | 0.003084 | 0.00016 | **1.20×10^-6^** |
|  |  | 0.1 | 0.014199 | 0.003108 | 0.000136 | **4.91×10^-6^** |
|  |  | 0.5 | 0.016294 | 0.003158 | 0.000187 | **2.48×10^-7^** |
|  |  | 1 | 0.01561 | 0.003164 | 0.000167 | **8.11×10^-7^** |
|  | Type 2 Diabetes | 0.01 | -0.00313 | 0.003014 | 9.67×10^-6^ | 0.298869 |
|  |  | 0.05 | -0.0015 | 0.003032 | 2.21×10^-6^ | 0.619643 |
|  |  | 0.1 | -0.00228 | 0.003041 | 5.03×10^-6^ | 0.453812 |
|  |  | 0.5 | 0.000433 | 0.003069 | 1.78×10^-7^ | 0.887774 |
|  |  | 1 | 0.001598 | 0.00307 | 2.43×10^-6^ | 0.602796 |

**Supplementary Table 3a**, Associations between polygenic profiles of health related traits, and neuroticism controlling for age, assessment centre, genotyping batch and array, and ten principal components for population structure stratified by gender. Statistically significant values are shown in bold.

|  |  | **Females (n = 57,074)** | | | **Males (n = 50,964)** | | |
| --- | --- | --- | --- | --- | --- | --- | --- |
| **Trait Category** | **Traits from GWAS consortia** | **β** | **r2** | **p** | **β** | **r2** | **p** |
| **Mental health** | ADHD | 0.0059 | 3.50E-05 | 0.1554 | 0.0031 | 1.02E-05 | 0.4675 |
|  | Alzheimer's Disease | -0.0055 | 3.04E-05 | 0.1855 | -0.0076 | 5.87E-05 | 0.0817 |
|  | Anorexia Nervosa | 0.0004 | 1.42E-07 | 0.9280 | 0.0108 | 0.0001 | **0.0126** |
|  | Bipolar Disorder | 0.0152 | 0.00023 | **0.0003** | 0.0193 | 0.0004 | **1.06E-05** |
|  | Borderline Personality | 0.0146 | 0.00021 | **0.0005** | 0.0156 | 0.0003 | **2.78E-04** |
|  | Major Depressive Disorder | 0.0373 | 0.00134 | **1.54E-18** | 0.0340 | 0.0012 | **1.04E-14** |
|  | Negative Affect (anxiety) | 0.0112 | 0.00013 | **0.0065** | 0.0074 | 5.65E-05 | 0.0877 |
|  | Neuroticism (GPC) | 0.0448 | 0.00197 | **5.29E-27** | 0.0417 | 0.0017 | **3.59E-22** |
|  | Schizophrenia | 0.0309 | 0.00092 | **3.44E-13** | 0.0417 | 0.0017 | **3.23E-21** |
|  |  |  |  |  |  |  |  |
| **Physical health** | Blood Pressure: Diastolic | -0.0091 | 8.26E-05 | 0.0290 | 4.68E-05 | 2.28E-09 | 0.9914 |
|  | Blood Pressure: Systolic | -0.0059 | 3.49E-05 | 0.1556 | 0.0032 | 1.07E-05 | 0.4584 |
|  | BMI | -0.0070 | 4.87E-05 | 0.0939 | -0.0123 | 0.0002 | **0.0043** |
|  | Coronary Artery Disease | 0.0117 | 0.00014 | **0.0048** | 0.0100 | 0.0001 | **0.0212** |
|  | Longevity | -0.0051 | 2.59E-05 | 0.2214 | -0.0051 | 2.65E-05 | 0.2424 |
|  | Rheumatoid Arthritis | 0.0001 | -6.99E-05 | 0.9734 | -0.0016 | 2.71E-05 | 0.7071 |
|  | Smoking Status | 0.0211 | 3.31E-04 | **1.53E-06** | 0.0107 | 1.32E-04 | **0.0183** |
|  | Type 2 Diabetes | 0.0010 | 9.47E-07 | 0.8152 | -0.0077 | 6.08E-05 | 0.0765 |

**Supplementary Table 3b**, Associations between polygenic profiles of health related traits, and neuroticism controlling for sex, assessment centre, genotyping batch and array, and ten principal components for population structure stratified by age (under and over 60 years). Statistically significant values are shown in bold.

|  |  | **Under 60 years (n = 65 337)** | | | **Over 60 years (n = 42 701)** | | |
| --- | --- | --- | --- | --- | --- | --- | --- |
| **Trait Category** | **Traits from GWAS consortia** | **β** | **r2** | **p** | **β** | **r2** | **p** |
| **Mental health** | ADHD | 0.0058 | 3.26E-05 | 0.1403 | 0.0033 | 1.2E-05 | 0.4648 |
|  | Alzheimer's Disease | -0.0067 | 4.30E-05 | 0.0905 | -0.0065 | 4.6E-05 | 0.1561 |
|  | Anorexia Nervosa | 0.0041 | 1.57E-05 | 0.3057 | 0.0072 | 5.7E-05 | 0.1154 |
|  | Bipolar Disorder | 0.0205 | 0.0004 | **3.12E-07** | 0.0119 | 0.0002 | **0.0097** |
|  | Borderline Personality | 0.0164 | 0.0003 | **3.77E-05** | 0.0132 | 0.0002 | **0.0036** |
|  | Major Depressive Disorder | 0.0390 | 0.0014 | **4.59E-22** | 0.0315 | 0.0011 | **1.1E-11** |
|  | Negative Affect (anxiety) | 0.0144 | 0.0002 | **0.0003** | 0.0024 | 6.2E-06 | 0.6024 |
|  | Neuroticism (GPC) | 0.0440 | 0.0019 | **9.81E-29** | 0.0425 | 0.0020 | **1.7E-20** |
|  | Schizophrenia | 0.0385 | 0.0014 | **2.17E-21** | 0.0324 | 0.0011 | **3.2E-12** |
|  |  |  |  |  |  |  |  |
| **Physical health** | Blood Pressure: Diastolic | -0.0006 | 3.17E-07 | 0.8844 | -0.0117 | 0.0001 | **0.01061** |
|  | Blood Pressure: Systolic | 0.0016 | 2.33E-06 | 0.6934 | -0.0068 | 5.2E-05 | 0.1338 |
|  | BMI | -0.0104 | 0.0001 | **0.0087** | -0.0081 | 7.2E-05 | 0.0768 |
|  | Coronary Artery Disease | 0.0094 | 8.34E-05 | **0.0184** | 0.0135 | 0.0002 | **0.0029** |
|  | Longevity | -0.0064 | 3.84E-05 | 0.1096 | -0.0036 | 1.4E-05 | 0.4321 |
|  | Rheumatoid Arthritis | -0.0008 | -4.64E-05 | 0.8382 | 5.63E-04 | 5.5E-06 | 0.9027 |
|  | Smoking Status | 0.0180 | 2.34E-04 | **1.53E-05** | 1.35E-02 | 0.0002 | **0.0051** |
|  | Type 2 Diabetes | -0.0021 | 4.31E-06 | 0.5918 | -0.0043 | 2.1E-05 | 0.3442 |

**Supplementary Table 3c**, Associations between polygenic profiles of health related traits, and neuroticism controlling for assessment centre, genotyping batch and array, and ten principal components for population structure stratified by gender and age (females under and over 60 years). Statistically significant values are shown in bold.

|  |  | **Females under 60 (n = 35 774)** | | | **Females over 60 (n = 21 300)** | | |
| --- | --- | --- | --- | --- | --- | --- | --- |
| **Trait Category** | **Traits from GWAS consortia** | **β** | **r2** | **p** | **β** | **r2** | **p** |
| **Mental health** | ADHD | 0.0060 | 3.58E-05 | 0.2576 | 0.0065 | 4.56E-05 | 0.3239 |
|  | Alzheimer's Disease | -0.0043 | 1.80E-05 | 0.4224 | -0.0081 | 7.3E-05 | 0.2122 |
|  | Anorexia Nervosa | -0.0013 | 1.59E-06 | 0.8112 | 0.0035 | 1.28E-05 | 0.6016 |
|  | Bipolar Disorder | 0.0210 | 0.0004 | **0.0001** | 0.0052 | 2.89E-05 | 0.4325 |
|  | Borderline Personality | 0.0140 | 0.0002 | **0.0097** | 0.0157 | 0.000267243 | **0.0170** |
|  | Major Depressive Disorder | 0.0396 | 0.0015 | **5.09E-13** | 0.0351 | 0.0013 | **1.64E-07** |
|  | Negative Affect (anxiety) | 0.0192 | 0.0004 | **0.0003** | -0.0016 | 2.57E-06 | 0.8149 |
|  | Neuroticism (GPC) | 0.0435 | 0.0019 | **4.10E-16** | 0.0463 | 0.0023 | **3.23E-12** |
|  | Schizophrenia | 0.0335 | 0.0010 | **9.79E-10** | 0.0276 | 0.0008 | **3.81E-05** |
|  |  |  |  |  |  |  |  |
| **Physical health** | Blood Pressure: Diastolic | -0.0038 | 1.38E-05 | 0.4814 | -0.0195 | 0.0004 | **0.0031** |
|  | Blood Pressure: Systolic | -0.0025 | 6.07E-06 | 0.6412 | -0.0121 | 0.0002 | 0.0659 |
|  | BMI | -0.0040 | 1.53E-05 | 0.4596 | -0.0109 | 0.0001 | 0.0999 |
|  | Coronary Artery Disease | 0.0112 | 0.0001 | **0.0361** | 0.0113 | 0.0001 | 0.0846 |
|  | Longevity | -0.0065 | 4.02E-05 | 0.2305 | -0.0038 | 1.49E-05 | 0.5734 |
|  | Rheumatoid Arthritis | 0.0035 | -7.98E-06 | 0.5140 | -0.0040 | 8.23E-06 | 0.5435 |
|  | Smoking Status | 0.0244 | 5.05E-04 | **1.48E-05** | 0.0156 | 0.0002 | **0.0251** |
|  | Type 2 Diabetes | -0.0024 | 5.54E-06 | 0.6560 | 0.0072 | 5.65E-05 | 0.2723 |

**Supplementary Table 3c**, Associations between polygenic profiles of health related traits, and neuroticism controlling for assessment centre, genotyping batch and array, and ten principal components for population structure stratified by gender and age (males under and over 60 years). Statistically significant values are shown in bold.

|  |  | **Males under 60 years (n = 29,563)** | | | **Males over 60 years (n = 21,401)** | | |
| --- | --- | --- | --- | --- | --- | --- | --- |
| **Trait Category** | **Traits from GWAS consortia** | **β** | **r2** | **p** | **β** | **r2** | **p** |
| **Mental health** | ADHD | 0.0057 | 3.15E-05 | 0.3348 | 0.0005 | 2.99E-07 | 0.9363 |
|  | Alzheimer's Disease | -0.0098 | 9.28E-05 | 0.0976 | -0.0048 | 2.68E-05 | 0.4487 |
|  | Anorexia Nervosa | 0.0106 | 0.0001 | 0.0730 | 0.0105 | 0.0001 | 0.0937 |
|  | Bipolar Disorder | 0.0200 | 0.0004 | **0.0008** | 0.0191 | 0.0004 | **0.0028** |
|  | Borderline Personality | 0.0194 | 0.0004 | **0.0010** | 0.0110 | 0.0001 | 0.0811 |
|  | Major Depressive Disorder | 0.0385 | 0.0014 | **1.28E-10** | 0.0280 | 0.0009 | **1.36E-05** |
|  | Negative Affect (anxiety) | 0.0083 | 6.64E-05 | 0.1612 | 0.0065 | 4.79E-05 | 0.3111 |
|  | Neuroticism (de Moor, 2015) | 0.0446 | 0.0019 | **3.31E-14** | 0.0388 | 0.0017 | **7.70E-10** |
|  | Schizophrenia | 0.0448 | 0.0019 | **1.17E-13** | 0.0372 | 0.0016 | **6.73E-09** |
|  |  |  |  |  |  |  |  |
| **Physical health** | Blood Pressure: Diastolic | 0.0032 | 1.01E-05 | 0.5853 | -0.0044 | 2.30E-05 | 0.4830 |
|  | Blood Pressure: Systolic | 0.0065 | 4.12E-05 | 0.2699 | -0.0019 | 4.03E-06 | 0.7689 |
|  | BMI | -0.0180 | 0.0003 | **0.0021** | -0.0053 | 3.22E-05 | 0.4062 |
|  | Coronary Artery Disease | 0.0070 | 4.68E-05 | 0.2394 | 0.0156 | 0.0003 | **0.0135** |
|  | Longevity | -0.0062 | 3.71E-05 | 0.2946 | -0.0037 | 1.56E-05 | 0.5629 |
|  | Rheumatoid Arthritis | -0.0059 | 3.16E-05 | 0.3174 | 0.0051 | 3.93E-05 | 0.4254 |
|  | Smoking Status | 0.0101 | 8.79E-05 | 0.1027 | 0.0117 | 1.51E-04 | 0.0815 |
|  | Type 2 Diabetes | -0.0018 | 3.03E-06 | 0.7647 | -0.0158 | 0.0003 | 0.0132 |
